# Colorectal cancer incidence before and during the COVID-19 pandemic: a non-linear interrupted time-series analysis

**DOI:** 10.1038/s41598-026-51701-w

**Published:** 2026-05-25

**Authors:** Nina Grundmann, Amir Hakimhashemi, Sven Voigtländer, Martin Meyer, Jacqueline Müller-Nordhorn

**Affiliations:** Bavarian Cancer Registry, Bavarian Health and Food Safety Authority, Schweinauer Hauptstraße 80, 90441 Nuremberg, Germany

**Keywords:** COVID-19 pandemic, Colorectal cancer, Interrupted time-series analysis, Prediction, Incidence, Cancer registry data, Gastroenterology, Oncology

## Abstract

The COVID-19 pandemic affected healthcare worldwide. This study analysed age-standardised incidence rates (ASIR) of colorectal cancer (CRC) in Bavaria to assess the COVID-19 pandemic’s impact. We modelled CRC ASIR, overall and in the subgroups (sex, age group, site and stage), in the pre-pandemic (01/2007 to 02/2020) and pandemic (03/2020 to 02/2022) periods using interrupted time-series analyses. Predictions for the third year of the pandemic (03/2022 to 02/2023) were compared to expected ASIR. Comparing expected and observed CRC ASIR during the first year of the pandemic showed a significant relative change of -8.8% (95% CI: -12.7% to -4.6%), improving to 1.4% (95% CI: -4.5% to 7.2%) in the third year of the pandemic. Likewise, ASIR significantly decreased across almost all subgroups during the first year of the pandemic, recovering during the second and the third year of the pandemic. Early stages showed a significant estimated reduction, persisting until the third year of the pandemic (-4.7%; 95% CI: -8.6% to -0.9%). In contrast, for women aged 70 years and above and women with colon cancer, ASIR experienced a significant excess. In the first year of the pandemic, estimated CRC ASIR experienced significant decline, returning to the expected rates during the third year of the pandemic, for most, but not all subgroups. The relative difference remained significantly negative for early stages. A significant catch-up effect occurred in the subgroups of women aged 70 years and above and women with colon cancer. Overall, estimations indicate a recovery of CRC ASIR.

**Supplementary Information:**

The online version contains supplementary material available at 10.1038/s41598-026-51701-w.

## Introduction

The COVID-19 pandemic adversely affected healthcare worldwide. Healthcare prioritisations and legal restrictions in response to the acute increase in the number of patients requiring medical care and the subsequent reallocation of resources led to a restriction of the access to healthcare. These included the deferral of routine diagnostic procedures, the postponement of non-urgent clinical visits, the cancellation of elective surgeries and the temporary suspension of cancer screening programs^1–4^. With respect to cancer care, early detection was gravely hampered. Some studies suggested that substantial increases in the number of avoidable cancer deaths were to be expected as a result of diagnostic and therapeutic delays^2,5–9^. Though not shown yet, a stage shift may occur with more patients being diagnosed with an advanced-stage cancer with a worse prognosis. For example, a study from Italy indicated a shift towards forms with greater lymph node involvement for lung cancer^10^.

A significant decline in cancer diagnoses during the pandemic was reported worldwide and for different cancer sites^1,3,8,11–16^, suggesting a backlog of missing cases^13^, caused by either not yet detected or not yet reported cases. The COVID-19 pandemic was assumed to affect particularly cancers often detected at screening such as colorectal cancer (CRC). In Germany, a 10% decrease of incident cases of CRC in the pandemic period was reported^17^. In Bavaria, Germany’s second most populous state, colon cancer cases declined by 12% during the first year of the pandemic (Mach 2020 to February 2021), while rectal cancer cases decreased by 15%^18^. The decreases occurred particularly in early-stage CRC. The decline in cancer cases was especially pronounced in the two lockdown periods from 21st March until 3rd May 2020 (first lockdown) and from 9th December 2020 until 7th March 2021 (second)^18^.

Effective measures for early detection of CRC are available in Germany, for example, colonoscopy has been part of the national screening programme since 2002^19^. The detection of precancerous lesions and early-stage CRC may prevent the development of advanced cancer stages. The decrease of early-stage CRC during the pandemic may thus require increased preventive efforts. Identifying population groups at risk might lead to more focused interventions. In this study, we used interrupted time-series analysis to model the impact of the COVID-19 pandemic on CRC incidence in Bavaria during the different years of the pandemic. Furthermore, we analysed the impact by stratifying for sex, age group, cancer site and stage.

## Methods

### Data

This observational study of routine anonymized registry data was deemed not to require formal Ethics Committee approval by the Bavarian State Chamber of Physicians (reference number: 2025 − 1156). According to § 15 of the professional code of physicians of Bavaria retrospective reviews of anonymized patient data do not require ethical approval. For such studies a written Informed Consent from study participants is not required. This study focussed on malignant incident cases of CRC (International Statistical Classification of Diseases and Related Health Problems, Tenth Revision (ICD-10)^20^, codes C18-C20) diagnosed between January 2007 and February 2022 in Bavaria, Germany. Data were retrieved from the population-based Bavarian Cancer Registry (date of access: 15th November 2024). Being established in 1998, the Bavarian Cancer Registry registers all cancer cases in Bavaria reported by inpatient and outpatient facilities^21^. For CRC, completeness of cases was ascertained for whole Bavaria between 2007 and 2022. Completeness of coverage represents the degree to which all expected incident cases of cancer in the population are reported to the cancer registry, and is a data quality measure of cancer registries^22^. The ratio of death certificate only (DCO) cases to all reported cases, which ascertains the data quality^22^, was below 10% in Bavaria during the study period. DCO cases were included in the analyses. We analysed CRC cases stratified by demographic characteristics (sex and age group) and by tumour characteristics (cancer site and stage) as well as by period (01/2007-02/2020 and 03/2020-02/2022). CRC cases were stratified based on the Classification of the Union for International Cancer Control (UICC) as early (UICC I and UICC II) and advanced stage (UICC III and UICC IV) cancer.

According to the pandemic periods, we defined the following terms for this study: “*year prior to the pandemic*” for the period from March 2019 to February 2020; “*first year of the pandemic*” for the period from March 2020 to February 2021; “*second year of the pandemic*” for March 2021 to February 2022; and “*third year of the pandemic*” for March 2022 to February 2023 (prediction of the incidence rate, no observed data)^23^.

### Statistical methods

We calculated the monthly age-standardised incidence rates (ASIR) per 100,000 and the corresponding 95% confidence intervals (CI) for CRC in Bavaria, stratified by sex, age, cancer site and cancer stage, using the average Bavarian population of the respective year and the European standard population^24^. We aimed to model the trend of monthly CRC ASIR during the study period. We applied an interrupted time-series (ITS) method using a non-linear generalised additive mixed model (GAMM)^25^ with random components for monthly effects. Furthermore, we analysed the residuals of the GAMM model using an auto-correlation function^26^ to investigate, whether the residuals were auto-correlated and of which degree, i.e. lag. If the residuals were auto-correlated, we added auto-regressive (AR) components of the corresponding lag to modify the GAMM model for each individual case with auto-correlated residuals. Furthermore, we tested if the contribution of a moving-average component could improve the goodness of fit of the model according to the Bayesian Information Criterion (BIC). The specific analyses were performed for UICC cancer stage, for sex, for sex and age group, and for sex and cancer site. Moreover, to ensure the compatibility of hierarchical models stratified by sex, cancer site and cancer stage, we included a correction variable into the respective models. This variable was derived from the complete CRC ASIR data as well as the predictions of the corresponding model. In this study, two major model categories were established: pre-pandemic models and pandemic models. The pre-pandemic models were calibrated using data exclusively from the pre-pandemic period (January 2007 to February 2020). Using the pre-pandemic models, we predicted the expected ASIR for the three pandemic periods. In contrast, the pandemic models used data spanning both the pre-pandemic and the pandemic periods (January 2007 to February 2022). Using the pandemic models, we predicted the estimated ASIR for the third year of the pandemic (March 2022 to February 2023). We measured the relative differences between the expected ASIR (predicted by the pre-pandemic model) and the observed ASIR for the first and the second year of the pandemic. For the third year of the pandemic, the relative differences were calculated by comparing the expected and the estimated (predicted by the pandemic model) ASIR. For calculating the relative changes and the corresponding CIs, random monthly effects were filtered, as the analysis focussed on entire annual trends rather than monthly variations. To validate the reference model, a validation model was trained using CRC ASIR between January 2007 and December 2018 and validated using the data from January 2019 to December 2019 (Supplementary Fig. 1). All statistical analyses were performed with R (R Foundation for Statistical Computing), version 4.4.1.

## Results

Data of 136,161 incident malignant cases of CRC in Bavaria in the period between January 2007 and February 2022 were identified from the Bavarian Cancer Registry database. Table 1 presents the characteristics of cancer cases included. The percentage of microscopic confirmations accounted for 91.9%. The proportion of death certificate only (DCO) cases was 6.9%. More than half of all CRC cases were men (56.8%). Only 5.9% of cases were aged below 50 years. The average monthly colorectal cancer incidence in the year prior to the pandemic, as well as in the first and the second year of the pandemic for different subgroups is shown in Supplementary Table 1. Table 2 shows the average monthly CRC ASIR, overall and stratified by cancer stage, sex, sex and age group, and sex and cancer site, across the three periods of the pandemic. Table 3 presents relative differences between observed, estimated and expected CRC ASIR and the corresponding CIs (without monthly changes), in different pandemic periods and for different subgroups. The relative changes and the corresponding CIs are the major focus of our analyses.

**Table 1:** Characteristics of incident colorectal cancer cases in Bavaria, Germany (01/2007–02/ 2022).

	Male		Female		All	
	Absolute numbers *N*	Percent (%)	Absolute numbers *N*	Percent (%)	Absolute numbers *N*	Percent (%)
Cases N (%)	77,395	56.8	58,747	43.2	136,161	
*Microscopically verified*	72,231	93.3	52,824	89.9	125,074	91.9
*DCO cases*	4275	5.5	5169	8.8	9444	6.9
Age at diagnosis (years)						
0–39	1109	1.4	1144	2.0	2255	1.6
40–49	3212	4.1	2513	4.3	5729	4.2
50–59	10,928	14.1	6878	11.7	17,810	13.1
60–69	19,778	25.6	10,950	18.6	30,729	22.6
70–79	25,834	33.4	17,149	29.2	42,989	31.6
80+	16,534	21.4	20,113	34.2	36,649	26.9
UICC stage						
I	13,849	17.9	9815	16.7	23,669	17.4
II	16,773	21.7	13,030	22.2	29,805	21.9
III	17,769	23.0	12,981	22.1	30,753	22.6
IV	14,430	18.6	10,249	17.4	24,681	18.1
X (unknown)	14,574	18.8	12,672	21.6	27,253	20.0
Anatomical site (ICD-10)						
Colon (C18 incl. C18.1)	48,141	62.2	41,521	70.7	89,674	65.9
Rectum (C19/C20)	29,254	37.8	17,226	29.3	46,487	34.1
Grading						
Low (G1 or G2)	56,864	73.5	39,351	67.0	96,229	70.7
High (G3 or G4)	11,746	15.2	10,729	18.3	22,476	16.5
Missing	8782	11.3	8667	14.7	17,451	12.8

*CRC* colorectal cancer, *COVID-19* Coronavirus disease, *DCO* death certificate only, *G* grade, *ICD-10* International Statistical Classification of Diseases and Related Health Problems, Tenth Revision, *UICC* Union for International Cancer Control;

Well differentiated tumours (G1) and moderately differentiated tumours (G2) were defined as “low grade” tumours.

Poorly differentiated tumours (G3) and undifferentiated tumours (G4) were defined as “high grade” tumours (Wittekind C. (2020): TNM Classification of Malignant Tumours, Eighth Edition, Wiley, Weinheim, Germany).

**Table 2:** Observed, estimated and expected CRC average monthly ASIR ^a^ in Bavaria, Germany, in the period of 03/2020 to 02/2021 (first year of the pandemic), in the period of 03/2021 to 02/2022 (second year of the pandemic) and in the period of 03/2022 to 02/2023 (third year of the pandemic).

Characteristics	03/2020 to 02/2021 (95% CI)		03/2021 to 02/2022 (95% CI)		03/2022 to 02/2023 (95% CI)	
	Observed	Expected (pre-pandemic model)	Observed	Expected (pre-pandemic model)	Estimated (pandemic model)	Expected (pre-pandemic model)
Overall						
CRC	2.90 (2.66, 3.15)	3.18 (3.04, 3.32)	3.10 (2.85, 3.35)	3.06 (2.92, 3.21)	2.98 (2.81, 3.16)	2.94 (2.79, 3.10)
Cancer stage – UICC						
Early stage (I & II)	1.09 (10.94, 1.24)	1.25 (1.22, 1.28)	1.14 (1.00, 1.30)	1.20 (1.16, 1.23)	1.09 (1.05, 1.13)	1.15 (1.11, 1.18)
Advanced stage (III & IV)	1.23 (1.08, 1.40)	1.35 (1.29, 1.41)	1.25 (1.09, 1.42)	1.29 (1.20, 1.38)	1.19 (1.14, 1.25)	1.23 (1.12, 1.35)
Women						
All	2.28 (1.99, 2.60)	2.51 (2.46, 2.56)	2.50 (2.19, 2.83)	2.43 (2.37, 2.48)	2.42 (2.34, 2.50)	2.35 (2.29, 2.41)
Age groups (years)						
20–49	0.69 (0.38, 1.04)	0.82 (0.76, 0.89)	0.80 (0.46, 1.17)	0.84 (0.76, 0.91)	0.81 (0.70, 0.92)	0.85 (0.77, 0.94)
50–69	4.81 (3.85, 5.85)	5.37 (5.04, 5.69)	5.07 (4.09, 6.13)	5.33 (4.95, 5.70)	5.01 (4.48, 5.54)	5.28 (4.83, 5.74)
70+	13.18 (11.07, 15.48)	14.55 (13.75, 15.35)	14.73 (12.50, 17.16)	13.87 (13.04, 14.70)	14.28 (13.27, 15.29)	13.18 (12.31, 14.06)
Anatomical site (ICD-10)						
Colon (C18 incl. C18.1)	1.61 (1.37, 1.87)	1.73 (1.65, 1.76)	1.73 (1.48, 2.00)	1.63 (1.55, 1.71)	1.66 (1.61, 1.71)	1.56 (1.45, 1.67)
Rectum (C19/C20)	0.68 (0.52, 0.87)	0.78 (0.73, 0.82)	0.77 (0.60, 0.97)	0.74 (0.70, 0.78)	0.73 (0.69, 0.78)	0.71 (0.66, 0.75)
Men						
All	3.60 (3.23, 4.01)	3.94 (3.89, 4.00)	3.79 (3.40, 4.20)	3.78 (3.72, 3.84)	3.63 (3.54, 3.72)	3.61 (3.54, 3.67)
Age groups (years)						
20–49	0.79 (0.46, 1.16)	0.83 (0.79, 0.88)	0.87 (0.52, 1.26)	0.84 (0.79, 0.89)	0.87 (0.76, 0.98)	0.84 (0.78, 0.90)
50–69	7.90 (6.66, 9.23)	8.54 (8.03, 9.06)	7.95 (6.71, 9.28)	8.17 (7.61, 8.72)	7.57 (6.94, 8.20)	7.79 (7.19, 8.40)
70+	21.81 (18.73, 25.15)	24.56 (23.43, 25.70)	23.78 (20.55, 27.26)	23.41 (22.18, 24.63)	22.78 (21.20, 24.36)	22.25 (20.91, 23.59)
Anatomical site (ICD-10)						
Colon (C18 incl. C18.1)	2.26 (1.96, 2.58)	2.46 (2.41, 2.50)	2.34 (2.04, 2.66)	2.36 (2.31, 2.41)	2.25 (2.18, 2.32)	2.27 (2.21, 2.32)
Rectum (C19/C20)	1.35 (1.12, 1.61)	1.49 (1.44, 1.53)	1.45 (1.21, 1.72)	1.41 (1.36, 1.46)	1.38 (1.31, 1.45)	1.34 (1.28, 1.40)

*ASIR* age-standardised incidence rate, *CI* confidence interval, CRC colorectal cancer, *ICD-10* International Statistical Classification of Diseases and Related Health Problems, Tenth Revision, *UICC* Union Internationale Contre le Cancer (Union for International Cancer Control);

^a^ ASIR based on the Old European Standard Population; all rates are presented as cases per 100,000.

**Table 3:** Relative differences between observed, estimated and expected CRC average monthly ASIR ^a^ (in percent) in Bavaria, Germany, in the period of 03/2020 to 02/2021 (first year of the pandemic), in the period of 03/2021 to 02/2022 (second year of the pandemic) and in the period of 03/2022 to 02/2023 (third year of the pandemic).

Characteristics	03/2020 to 02/2021 Relative difference between observed and expected ASIR in percent (95% CI)	03/2021 to 02/2022 Relative difference between observed and expected ASIR in percent (95% CI)	03/2022 to 02/2023 Relative difference between estimated and expected ASIR in percent (95% CI)
Overall			
CRC	**-8.8 (-12.7**,** -4.6)**	1.1 (-3.5, 6.2)	1.4 (-4.5, 7.2)
Cancer stage – UICC			
Early stage (I & II)	**-13.1 (-15.0**,** -11.0)**	**-4.7 (-7.3**,** -2.1)**	**-4.7 (-8.6**,** -0.9)**
Advanced stage (III & IV)	**-8.5 (-12.4**,** -4.3)**	-3.4 (-9.5, 3.6)	-3.3 (-7.7, 1.1)
Women			
All	**-8.8 (-10.6**,** -7.0)**	**2.8 (0.5**,** 5.2)**	2.9 (-0.5, 6.3)
Age groups (years)			
20–49	**-16.1 (-22.4**,** -8.6)**	-4.8 (-12.6, 4.6)	-5.0 (-17.4, 7.3)
50–69	**-10.4 (-15.5**,** -4.7)**	-4.9 (-11.2, 2.4)	-5.1 (-15.1, 4.9)
70+	**-9.4 (-14.1**,** -4.2)**	**6.2 (0.2**,** 13.0)**	**8.3 (0.7**,** 16.0)**
Anatomical site (ICD-10)			
Colon (C18 incl. C18.1)	**-5.8 (-8.8**,** -2.5)**	**5.9 (0.8**,** 11.6)**	**6.7 (3.3**,** 10.1)**
Rectum (C19/C20)	**-12.5 (-16.8**,** -7.7)**	6.7 (-1.9, 9.8)	4.0 (-2.0, 10.1)
Men			
All	**-8.6 (-9.9**,** -7.4)**	0.3 (-1.2, 1.9)	0.6 (-1.8, 3.1)
Age groups (years)			
20–49	-4.8 (-9.9, 1.0)	4.5 (-1.4, 11.2)	3.7 (-9.1, 16.6)
50–69	**-7.6 (-12.8**,** -1.7)**	-2.7 (-8.9, 4.4)	-2.9 (-11.0, 5.2)
70+	**-11.2 (-15.1**,** -6.9)**	1.6 (-3.5, 7.2)	2.4 (-4.7, 9.5)
Anatomical site (ICD-10)			
Colon (C18 incl. C18.1)	**-8.1 (-9.8**,** -6.4)**	-1.1 (-3.2, 1.1)	-0.9 (-4.0, 2.2)
Rectum (C19/C20)	**-9.5 (-12.2**,** -6.6)**	2.7 (-0.9, 6.5)	3.2 (-2.0, 8.4)

*ASIR* age-standardised incidence rate, *CI* confidence interval, *CRC* colorectal cancer, *ICD-10* International Statistical Classification of Diseases and Related Health Problems, Tenth Revision, *UICC* Union for International Cancer Control;

^a^ ASIR based on the Old European Standard Population;

**Fig. 1:**
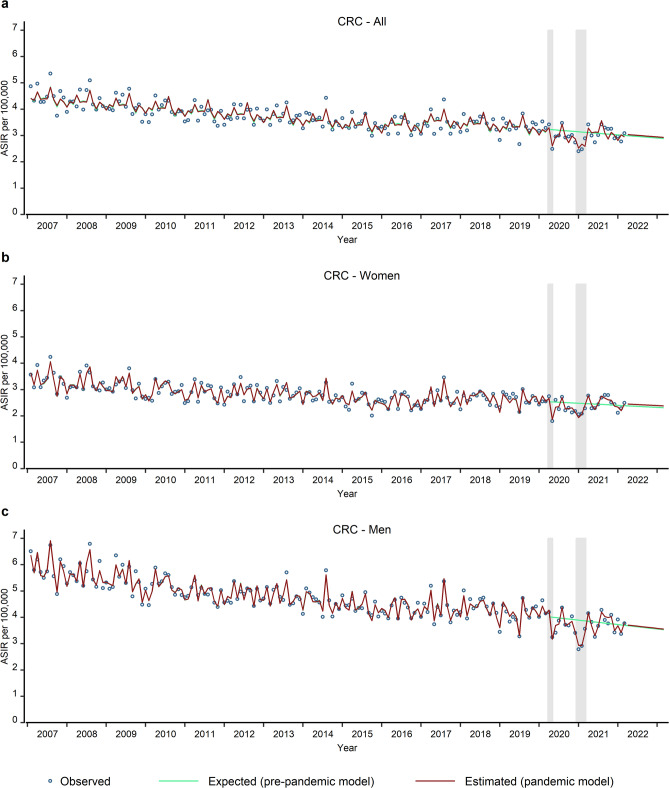
This figure shows the observed (blue circles), expected (pre-pandemic model, green curves) and estimated (pandemic model, red curves) monthly age-standardised incidence rates (ASIR) per 100,000 for CRC total (**a**), CRC in women (**b**) and CRC in men (**c**). The grey shaded areas indicate the COVID-19 pandemic lockdown periods in Bavaria. The green and red curves represent the fitted values. Additionally, the green curves present the predicted values (by the pre-pandemic model) from March 2020 to the end of the study period, while the red curves display the predicted values (by the pandemic model) from March 2022 to the end of the study period.

Comparing the observed average monthly CRC ASIR with the expected rate in the first year of the pandemic, revealed a significant relative change of -8.8% (95% CI: -12.7% to -4.6%) (Table 3; Fig. 1a), which improved during the second year (relative difference: 1.1%; 95% CI: -3.5% to 6.2%). The ASIR estimated for the third year of the pandemic indicated a return to the expected rate (relative difference: 1.4%; 95% CI: -4.5% to 7.2%). Stratified by sex, we found similar reductions for both, women and men (Fig. 1b and c) during the first year of the pandemic (Table 3). For women, a significant catch-up effect was detected in the second year (relative difference: 2.8%; 95%CI: 0.5% to 5.2%), but not significant in the third year of the pandemic.

**Fig. 2:**
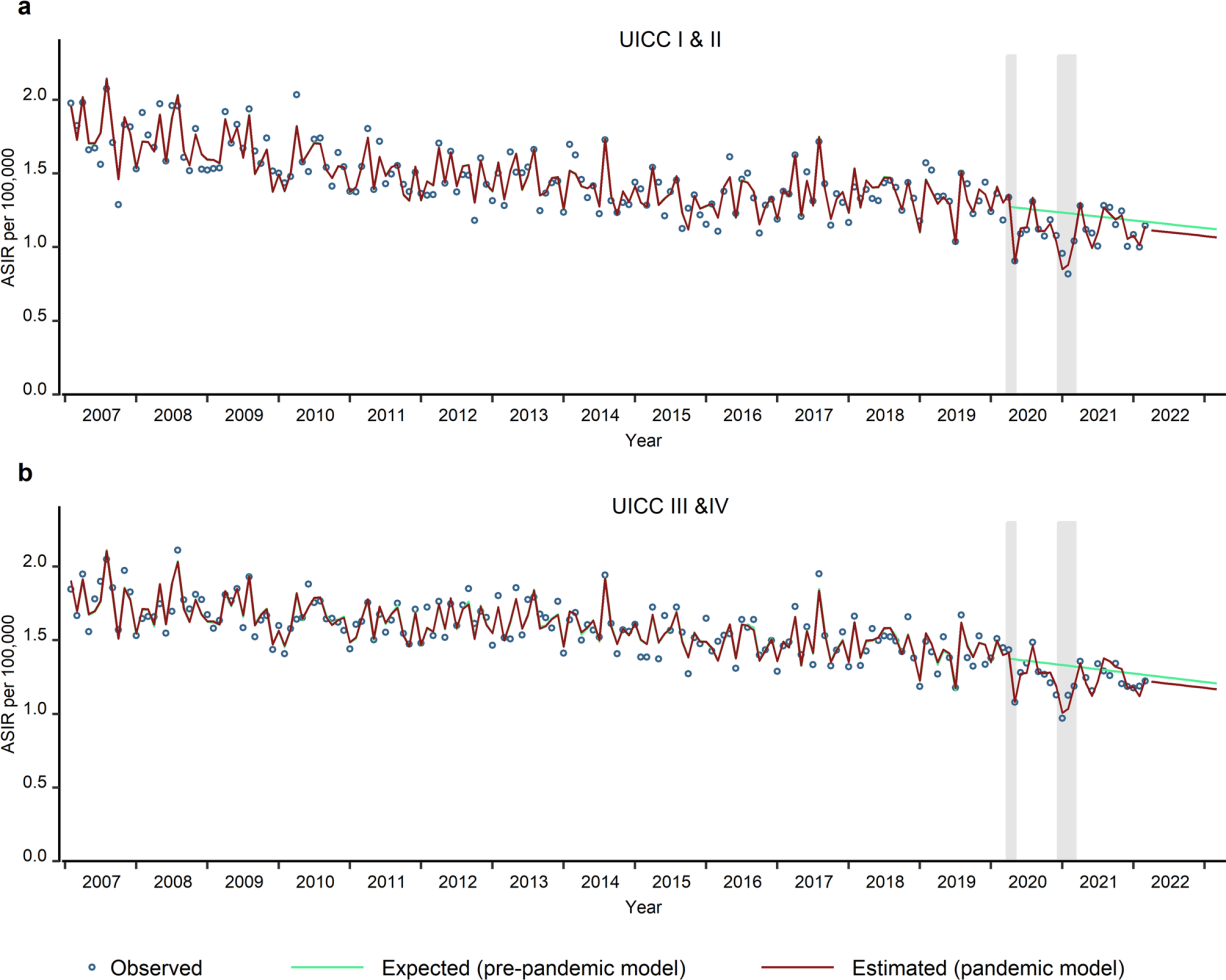
This figure shows the observed (blue circles), expected (pre-pandemic model, green curves) and estimated (pandemic model, red curves) monthly age-standardised incidence rates (ASIR) per 100,000 for CRC in Bavaria, stratified by cancer stage in early stages (UICC I&II) (**a**) and advanced stages (UICC III&IV) (**b**). The grey shaded areas indicate the COVID-19 pandemic lockdown periods in Bavaria. The green and red curves represent the fitted values. Additionally, the green curves present the predicted values (by the pre-pandemic model) from March 2020 to the end of the study period, while the red curves display the predicted values (by the pandemic model) from March 2022 to the end of the study period.

Stratified by cancer stage, we found significant reductions for both, early-stage cancer (relative difference: -13.1%; 95% CI: -15.0% to -11.0%) and advanced-stage cancer (relative difference: -8.5%; 95% CI: -12.4% to -4.3%) during the first year of the pandemic (Fig. 2a and b). The gap persisted significantly only for the early stages in the third year of the pandemic (relative difference: -4.7%; 95% CI: -8.6% to -0.9%). However, there were still a number of remaining cases with unknown stage, which have to be considered. In particular, the proportion of unknown stages increased from 22.7% in 2020 to 23.8% in 2021 and further to 26.1% in 2022.

**Fig. 3:**
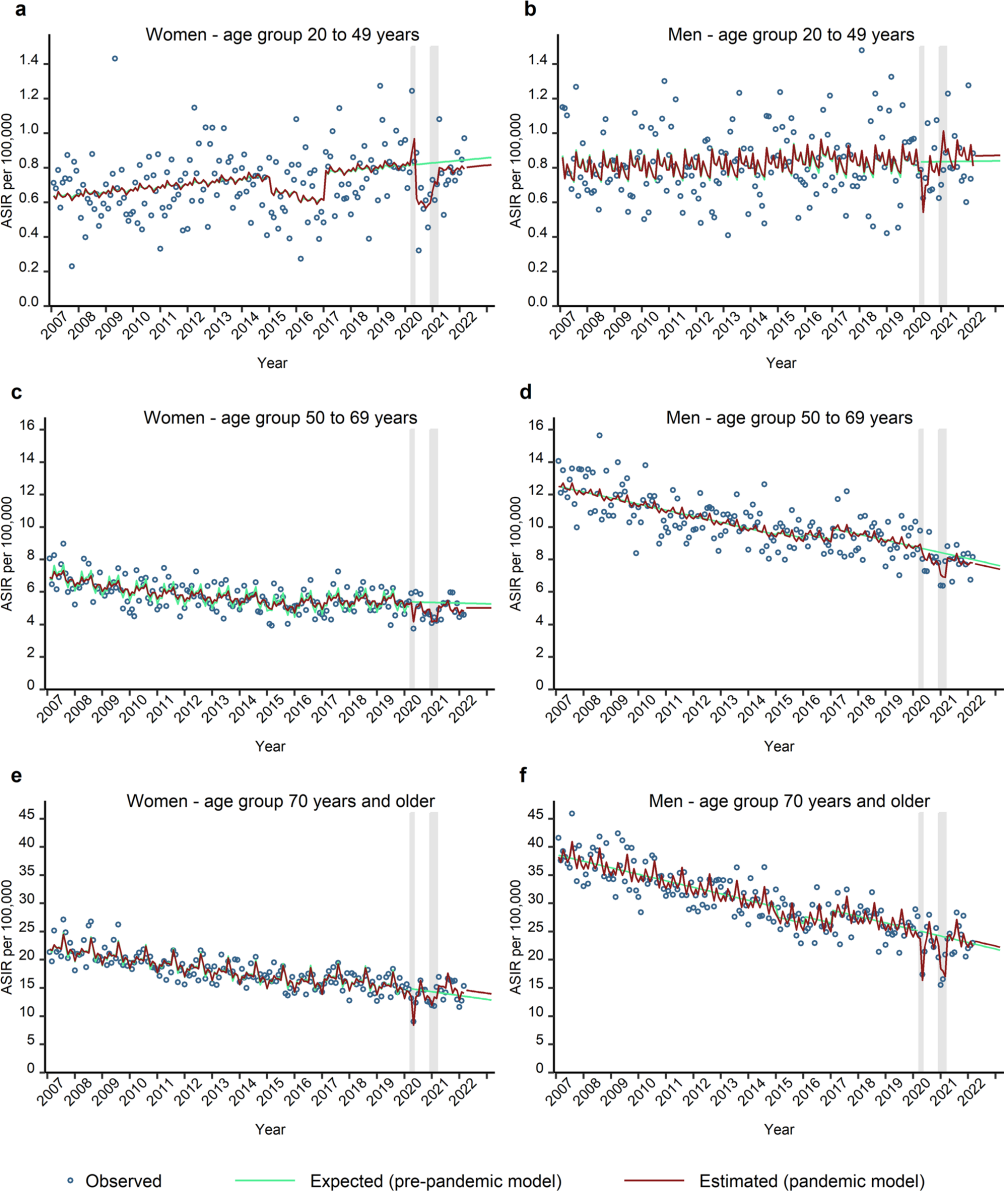
This figure shows the observed (blue circles), expected (pre-pandemic model, green curves) and estimated (pandemic model, red curves) monthly age-standardised incidence rates (ASIR) per 100,000 for CRC in Bavaria, stratified by sex and age group: age group 20–49 years: women (**a**), men (**b**); age group 50–69 years: women (**c**), men (**d**); age group 70 years and above: women (**e**), men (**f**). The grey shaded areas indicate the COVID-19 pandemic lockdown periods in Bavaria. The green and red curves represent the fitted values. Additionally, the green curves present the predicted values (by the pre-pandemic model) from March 2020 to the end of the study period, while the red curves display the predicted values (by the pandemic model) from March 2022 to the end of the study period.

**Fig. 4:**
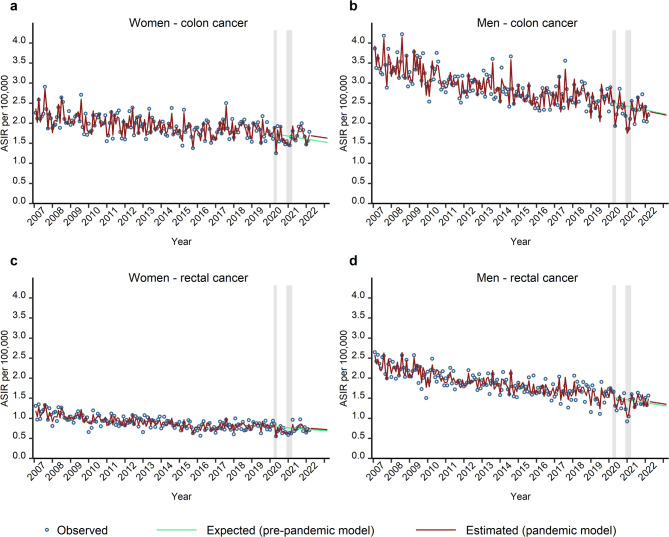
This figure shows the observed (blue circles), expected (pre-pandemic model, green curves) and estimated (pandemic model, red curves) monthly age-standardised incidence rates (ASIR) per 100,000 for CRC in Bavaria, stratified by sex and cancer site: colon cancer: women (**a**), men (**b**); rectal cancer: women (**c**), men (**d**). The grey shaded areas indicate the COVID-19 pandemic lockdown periods in Bavaria. The green and red curves represent the fitted values. Additionally, the green curves present the predicted values (by the pre-pandemic model) from March 2020 to the end of the study period, while the red curves display the predicted values (by the pandemic model) from March 2022 to the end of the study period.

Stratified by sex and age group (Fig. 3a–f), we found significant reductions in ASIR for all subgroups during the first year of the pandemic except for men aged 20 to 49 years (Table 3; Fig. 3b). The relative changes were not significant anymore during the second and the third year of the pandemic. Indeed, for women aged 70 years and above, a significant catch-up effect was detected in the second year (relative difference: 6.2%; 95% CI 0.2% to 13.0%) and in the third year of the pandemic (relative difference: 8.3%; 95% CI: 0.7% to 16.0%), respectively (Table 3; Fig. 3e). In men aged 70 years and above, the positive relative change was not significant, either during the second year or the third year of the pandemic (Table 3). Stratified by sex and cancer site, we detected significant reductions for both colon and rectal cancer in men and in women in the first year of the pandemic (Table 3; Fig. 4). ASIR for colon cancer in women showed a significant excess during the second year (relative difference: 5.9%; 95% CI: 0.8% to 11.6%) and the third year of the pandemic (relative difference: 6.7%; 95% CI: 3.3% to 10.1%) (Fig. 4a). However, there was no evidence of a significant catch-up in ASIR for colon cancer in men during the third year of the pandemic (Table 3; Fig. 4b). For rectal cancer in women and in men (Fig. 4c,d), the estimated ASIR exceeded the expected rates during the third year of the pandemic, however, the positive effect was not significant (Table 3).

## Discussion

In this study, we aimed to assess the impact of the COVID-19 pandemic for different subgroups of CRC to identify possible vulnerable groups and a potential need for action. We found a significant decrease in CRC incidence in all subgroups during the first year of the pandemic. Our analyses indicate that CRC ASIR largely returned to the expected rates in the third year of the pandemic. We detected a significant decline for both early and advanced stage CRC during the first year of the pandemic, which was more prominent for the early stages. The gap did not completely subside in the second and the third year of the pandemic. However, the reduction was significant only for early-stage CRC during the second and the third year of the pandemic. Nevertheless, we could not completely exclude a catch-up effect due to the increased proportion of CRC with unknown stage during the third year of the pandemic.

Our findings are in line with previous studies. A recovery of ASIR for most sites was also reported for the Netherlands^27^, Canada^28,29^ and the US^30^. Significant reductions in cancer incidence during the pandemic period were reported worldwide^13,31^. Reductions differed by cancer site. In Germany, the strongest reductions were found for CRC and prostate cancer followed by melanoma and breast cancer, the latter especially during the first wave of the pandemic^17^. In Italy, as well, a sharp drop in incidence has been observed, especially for melanoma and nonmelanoma skin cancer, CRC, prostate and bladder cancer^32^. In the US, the largest declines were observed for melanoma, lung and prostate cancer^33^, while decreases in the UK and the Nordic countries predominantly occurred for breast and prostate cancer^17,34,35^. Large reductions were also observed for breast cancer in the Netherlands^17^ and in Canada^28^. However, CRC was one of the cancer types with the largest decreases^29^ and significant declines in ASIR were found for both sites, the colon and the rectum^33^. Relating to all cancer sites, decreases were most pronounced in early-stage cancers^12,27,33^.

Investigating the effect of the pandemic on general and specialised practices in Germany, two studies reported significant declines in the number of incident cancer diagnoses in almost all age groups both, in men and in women. The declines were most prominent in individuals aged 70 years and above^1,36^. These findings are supported by our research. We found absolute reductions of CRC ASIR to be highest in the age group 70 years and above. This might reflect the mitigation measures recommended by the Federal State of Bavaria^36^. As higher age groups were at the highest risk for a serious COVID-19 infection, personal precautionary actions to avoid an infection may have played a role as well. Especially elderly people refrained from seeking medical care during the pandemic^37^. Finally, the incidence decrease found by different studies in the highest age group could also be attributed to the impact of COVID-19 on mortality^36,38^. Nevertheless, our analysis indicated a significant catch-up effect in the second and the third year of the pandemic for women aged 70 years and above, suggesting individuals of this age group have resumed regular medical check-ups and cancer screening. Having found significant reductions of annual ASIR for all age groups, even for individuals younger than 50 years, Johansson et al. hypothesised a lower healthcare utilisation in general during 2020 and 2021^35^. Corresponding to the middle age group, a study from Canada and the Nordic countries found the largest deficit of cases among individuals aged 50 to 65 and 50 to 69 years, respectively^29,35^. We also found a significant reduction of CRC ASIR for the age group 50 to 69 years during the first year of the pandemic, which continued, though not significant, in the subsequent years. However, there was no sign of recovery of CRC ASIR for individuals of this age group in the study period. The age group 50 to 74 years has been indicated as the target age group to offer CRC screening by the 2003 EU Council recommendations^39,40^. In Germany, the national screening programme offered up to two screening colonoscopies taken with a time distance of at least 10 years apart to men and women aged 55 years or older^2^. Starting age of screening colonoscopy was lowered from 55 to 50 years in July 2019, first only for men, and in April 2025, equally for women. As an alternative screening option the faecal occult blood test (FOBT) was offered, or respectively, since 2017, the immunological fecal occult blood test (iFOBT, FIT)^2^. Due to the evidence, that the risk of complications increases with age, screening colonoscopy is usually not performed in patients aged 75 years and above. If the first screening colonoscopy is performed in the age of 65 years or older, no second screening colonoscopy is included in the programme. However, there is no age limitation for iFOBT^41^. Some studies suggest that the incidence deficits in the age group 50 to 74 years might be the result of decreased participation in CRC screening and reductions in the availability of colonoscopies during the pandemic^28^. The hypothesis is supported by a recently published study from the Netherlands, which showed that the COVID-19 pandemic had the largest impact on CRC incidence among the age group 55 to 75 years^42^.

Some studies revealed differences in CRC ASIR during and after the pandemic stratified by age group and site. A study from Manitoba, Canada observed substantial reductions of ASIR for colon cancer in total during 1st April, 2020 to 31th December, 2021 (defined as the pandemic period), which was mainly driven by the age group 50 years and above, while no reduction was detected for individuals younger than 50 years^28^. For rectal cancer, the study found only a temporary decline in incidence rate in the age group 50 to 74^28^.

Our results show that CRC incidence rates experienced a continuous decline over the study period, which was more pronounced for early-stage CRC, likely indicating an effective CRC screening. Investigating CRC incidence trends in Europe, Cardoso et al. found decreasing incidence rates in countries which had already implemented CRC screening^43^. Investigating the impact of the COVID-19 pandemic on cancer stages, international studies mainly focussed on the first year of the pandemic. Large reductions were found especially for early-stage CRC^12,27,29,42^. Stage I CRCs, are generally asymptomatic and are more likely to be detected by screening, while the severity of symptoms caused by CRC stage IV initiate patients to seek medical care^44,45^. Correspondingly, one study from Canada found reductions in the number of CRC cases for UICC stage I to III, but not for stage-IV cancers^29^. For the Nordic countries, Denmark, Iceland, Norway, and Sweden, Johansson et al. observed significant reductions for colon cancer in 2020 only for stage I or/and stage II^35^.Another study from the Netherlands, claimed the largest decrease for stage I CRC, while a lower decline was found for stage II and III cancers and only a brief fall for stage IV among individuals of the age group 55 to 75 years^27^. The same study noted a catch-up effect in CRC overall, still in the first year of the pandemic, reporting that for stage III, observed CRC incidence was higher than the expected ASIR. As an explanation, the authors suggested that the increase was more likely due to enhanced screening capacity following its temporal suspension rather than a shift from early to advanced stages, though further monitoring was required^27^. Regarding a possible stage shift, a study from Quebec, Canada, suggested a possible shift for UICC stages I, II and III^29^. A study from the US found the largest decrease in stage I CRC, which was even more distinct for medically underserved individuals and those living in socioeconomically deprived areas^12^. We found substantial reductions in ASIR during the first year of the pandemic for both, early and advanced stage cancers, though the decrease was stronger in early stages. For the latter, the decrement persisted significantly until the end of the study period. Reductions in the total number of diagnostic and screening colonoscopies were likewise reported for Germany between 2020 and 2022 due to the pandemic^46^. Thus, the found reductions may indicate a possible deficit in early detection. However, the proportion of cases with unknown stage should be considered, when evaluating cancer stages during the pandemic phases. Finally, the decrease could be related to still unreported cases or even to patients who died undiagnosed during the pandemic period.

We also observed an increase of unstaged cancer since 2020 in our study (Supplementary Fig. 2). Several factors may contribute to this observation, including structural processes of cancer registration as well as the legal regulations (e.g., the demand of case completeness, i.e. minimum 90% of expected incident cancer cases have to be recorded for each year). For this reason, notifications of the diagnosis are given priority towards notifications with further information, e.g. on stage or decisions on treatment options, which are recorded afterwards. During the pandemic, the delay was additionally enhanced, since registration of cancer cases, and consequently data consolidation, was delayed due to legal restrictions and scarce resources. Moreover, the evaluation of the cancer by pathologists and the subsequent staging can only be performed after surgical intervention has taken place. These procedures, in themselves, prolong the time of staging since the first diagnosis and, of course, may also have been affected by the pandemic. Finally, while the proportion of unknown stage increased since 2020, the proportion of UICC stages I to IV started to decrease simultaneously (Supplementary Fig. 2). This indicates that the distribution of UICC stages I to IV probably did not change during the pandemic periods.

In this study, we used an ITS analysis with a long pre-intervention period including the two lockdown phases, accounting for multiple interventions as well as monthly effects to ensure highly reliable estimations. The ITS approach was also used by other studies investigating the impact of the COVID-19 pandemic on cancer incidence^28,29^. However, their analyses were limited up to the first year of the pandemic. One study from Quebec, Canada, suggested a possible stage shift in CRC, while the second study used cancer registry data from Manitoba, Canada to estimate the cumulative difference between the expected and the fitted ASIR during the pandemic, considering different cancer sites and age groups^28^. However, to our knowledge, our study is the only one using ITS models to predict cancer incidence in the third year of the pandemic.

## Strengths and limitations

Our study has several strengths. First, the population-based analysis ensures high representativeness of the data by reducing the selection bias. This, along with the use of quality-assured Cancer Registry data, enabled reliable assertions for Bavaria, which has more than 13 million inhabitants and is the second most populous federal state of Germany, exceeding the populations of countries such as Belgium, Sweden, and Portugal. Second, the longitudinal character of the data allowed a broad observation period, not limited to the narrow timeframe often used for the investigation of the impact of COVID-19 on cancer incidence. Another strength relates to our choice of the method. Using a generalised model for the estimation of CRC incidence based on monthly incidence rates ascertained the compatibility and goodness-of-fit of models, especially for the data of this study, where the stationarity could not be guaranteed. In general, applying stationary methods (like linear models) to non-stationary data including trends, seasonality (in our case monthly effects), auto-correlation, and breaks (interventions), leads to bias or misleading results. Furthermore, our study comprises, in addition to the first, also the second and the third year of the pandemic. The ITS models with several interventions, used in this study, had the capacity to predict the CRC incidence rates. By employing various statistical tests, e.g. autocorrelation test, we could confirm the high accuracy of our results. This is achieved by incorporating essential components into our models, such as random effects and autoregressive elements.

This study has some limitations that should be taken into account when interpreting the results. First, the most recent data available were limited to February 2022 due to the delayed completion of reporting of the data, e.g. cancer stage. The proportion of CRC with unknown stage increased in 2021 and 2022, which can affect the interpretation of the results according to both early and advanced stages.

## Conclusion

Our analysis suggests that the incidence of CRC experienced a significant decline during the first year of the pandemic, especially during the lockdown phases. Also, our findings indicate that CRC ASIR largely returned to the expected rates until the third year of the pandemic. However, a significant catch-up effect could not be detected for all subgroups. In particular, the gap between estimated and expected rates seemed to persist in early-stage cancers until the third year of the pandemic, which might indicate a possible deficit in early detection during the pandemic. Therefore, the continuation of early detection even in the pandemic situation should be a key issue for public health policy. Nevertheless, the trends estimated for CRC ASIR indicate a recovery of rates in the future.

## Supplementary Information

Below is the link to the electronic supplementary material.

Supplementary Material 1

## Data Availability

Following publication, the anonymized datasets generated and/or analysed during the current study are available from the corresponding author to researchers who provide a methodologically sound proposal, provided approval from the Advisory Board of the Bavarian Cancer Registry.
