# Supplementary material for: Colorectal cancer incidence before and during the COVID-19 pandemic: a non-linear interrupted time-series analysis
Source: Sci Rep. 2026 May 25;16:16147. doi: 10.1038/s41598-026-51701-w (PMC13201592; doi:10.1038/s41598-026-51701-w)
Supplement: Supplementary file 1 — Supplementary Material 1 [file 41598_2026_51701_MOESM1_ESM.docx]

**Supplement**

**
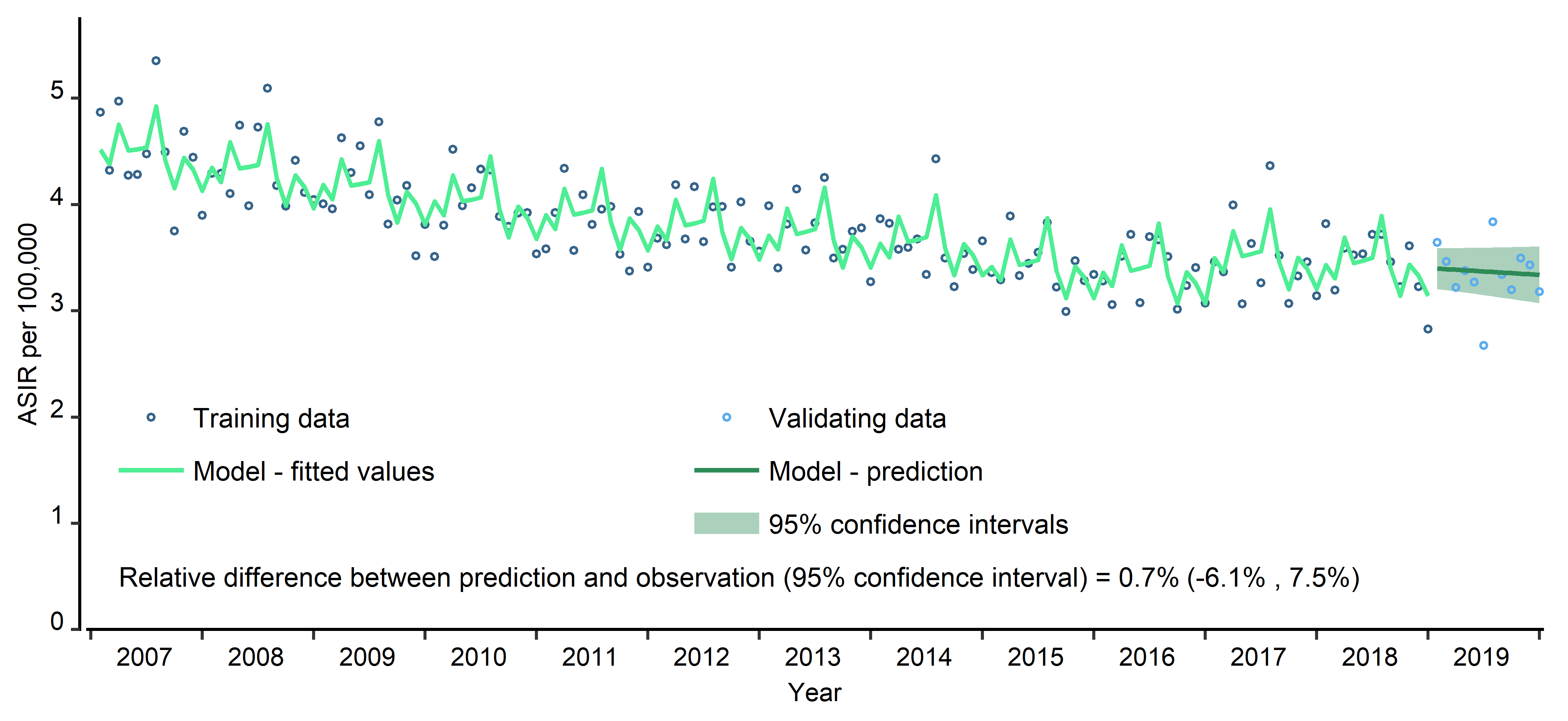
**

**Supplementary Figure 1:** This figure shows the validation model, which was trained using CRC ASIR between January 2007 and December 2018 (dark blue circles) and validated using the data from January 2019 to December 2019 (light blue circles). The light green curve represents the fitted values from the model using the training data, while the dark green curve and shaded area show the predicted values and the corresponding confidence intervals for the validation period.


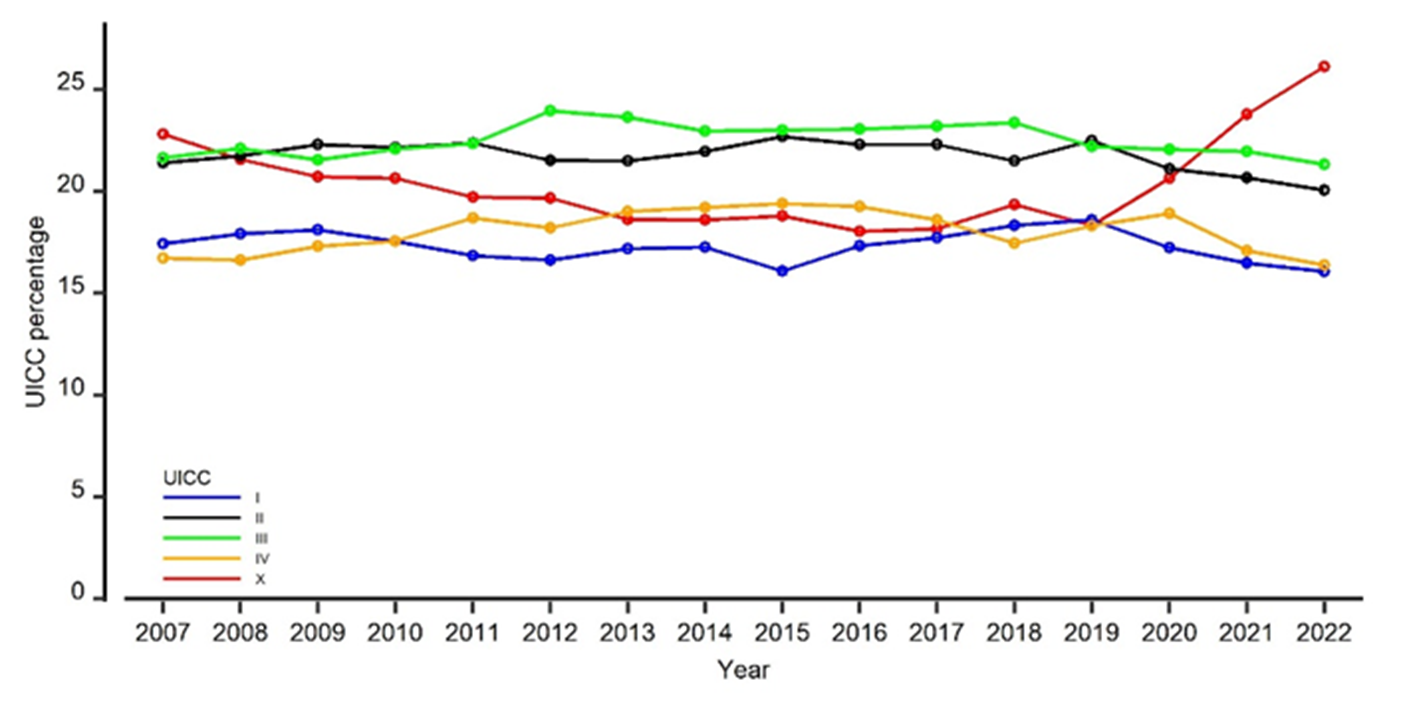


**Supplementary Figure 2:** This figure shows the proportion [Percentage] of UICC stages (I, II, III, IV, X) of CRC by year in Bavaria. The the proportion of unknown stages increased from 22.7% in 2020 to 23.8% in 2021 and further to 26.1% in 2022. Simultaneously, the proportion of the UICC stages I, II, III and IV decreased.

**Supplementary Table 1: Average monthly colorectal cancer incidence in Bavaria, Germany, in the period of 03/2019 to 02/2020 (year prior to** **the pandemic), in the period of 03/2020 to 02/2021 (first year of the pandemic) and in the period of 03/2021 to 02/2022 (second year of the pandemic)**

| **Rates** | **Women** | | | **Men** | | | **All** | | |
| --- | --- | --- | --- | --- | --- | --- | --- | --- | --- |
|  | **03/2019 – 02/2020** | **03/2020 – 02/2021** | **03/2021 – 02/2022** | **03/2019 – 02/2020** | **03/2020 – 02/2021** | **03/2021 – 02/2022** | **03/2019 – 02/2020** | **03/2020 – 02/2021** | **03/2021 – 02/2022** |
| *Overall incidence rate* |  |  |  |  |  |  |  |  |  |
| Crude | 4.67 | 4.09 | 4.50 | 6.36 | 5.55 | 5.85 | 5.51 | 4.82 | 5.17 |
| Age-standardised ^a^ | 2.61 | 2.27 | 2.47 | 4.12 | 3.58 | 3.75 | 3.31 | 2.89 | 3.07 |
|  |  |  |  |  |  |  |  |  |  |
| *Age-specific rates by age group, years ^a^* |  |  |  |  |  |  |  |  |  |
| 0–39 | 0.21 | 0.21 | 0.26 | 0.21 | 0.19 | 0.18 | 0.21 | 0.20 | 0.22 |
| 40–49 | 1.76 | 1.32 | 1.42 | 1.67 | 1.63 | 1.91 | 1.72 | 1.48 | 1.66 |
| 50–59 | 3.58 | 3.63 | 3.45 | 5.81 | 5.02 | 5.22 | 4.71 | 4.34 | 4.34 |
| 60–69 | 7.53 | 6.47 | 7.29 | 13.61 | 11.98 | 11.71 | 10.48 | 9.14 | 9.44 |
| 70–79 | 13.87 | 11.38 | 12.73 | 23.07 | 19.20 | 21.27 | 18.13 | 15.00 | 16.68 |
| 80+ | 20.21 | 17.40 | 19.09 | 31.92 | 27.71 | 29.03 | 24.68 | 21.32 | 22.89 |
| *Age-standardised rates by anatomical site (ICD-10) ^a^* |  |  |  |  |  |  |  |  |  |
| Colon (C18 incl. C18.1) | 1.79 | 1.60 | 1.71 | 2.57 | 2.24 | 2.31 | 2.15 | 1.89 | 1.99 |
| Rectum (C19/C20) | 0.82 | 0.68 | 0.76 | 1.56 | 1.34 | 1.44 | 1.17 | 0.99 | 1.08 |
| *Age-standardised rates by* *UICC-stage ^a^* |  |  |  |  |  |  |  |  |  |
| I | 0.48 | 0.43 | 0.43 | 0.74 | 0.59 | 0.62 | 0.60 | 0.50 | 0.52 |
| II | 0.57 | 0.46 | 0.49 | 0.86 | 0.72 | 0.75 | 0.70 | 0.58 | 0.61 |
| III | 0.58 | 0.50 | 0.54 | 0.95 | 0.83 | 0.88 | 0.75 | 0.66 | 0.70 |
| IV | 0.51 | 0.43 | 0.44 | 0.80 | 0.73 | 0.64 | 0.64 | 0.57 | 0.53 |
| X (unknown) | 0.48 | 0.46 | 0.56 | 0.76 | 0.74 | 0.89 | 0.61 | 0.59 | 0.71 |
| *Age-standardised rates by  tumour grading ^a^* |  |  |  |  |  |  |  |  |  |
| Low (1 or 2) | 1.90 | 1.68 | 1.79 | 3.12 | 2.68 | 2.82 | 2.47 | 2.14 | 2.27 |
| High (3 or 4) | 0.44 | 0.37 | 0.43 | 0.58 | 0.54 | 0.51 | 0.51 | 0.45 | 0.46 |
| Missing | 0.27 | 0.23 | 0.25 | 0.42 | 0.36 | 0.42 | 0.34 | 0.29 | 0.33 |

a) ASIR based on the Old European Standard Population; all rates are presented as cases per 100,000 person years,
Abbreviations: ICD-10 International Statistical Classification of Diseases and Related Health Problems, Tenth Revision; UICC, Union for International Cancer Control
